# 1542. MR-proADM Biomarker Predicts COVID-19 Clinical Outcomes: a US-based Cohort Study

**DOI:** 10.1093/ofid/ofac492.097

**Published:** 2022-12-15

**Authors:** Michael Mansour

**Affiliations:** Massachusetts General Hospital, Boston, Massachusetts

## Abstract

**Background:**

Mid-regional proadrenomedullin (MR-proADM) is a biomarker released following endothelial damage. Our study aimed to investigate baseline MR-proADM as a predictor of a wider range of clinical outcomes of varying severity in patients admitted with COVID-19 from a US-based multicenter trial.

Outcomes Within 28 days Stratified by Binary MR-proADM (Cutoff 0.87 Nmol/L), Number of Events (percentage)

Values expressed as percentages (%) indicate either the proportion of the total population or the respective MR-proADM stratum. Statistical significance between MR-proADM strata was determined by the chi-square test or the Fisher's exact test when applicable. P-Values were not corrected for multiple testing.

**Methods:**

Data from the Boston Area COVID-19 Consortium Bay Tocilizumab Trial (NCT04356937) was used in this study. Patients with biomarker determinations, and not admitted to the intensive care unit (ICU) on admission, were included. MR-proADM cutoff of 0.87 nmol/L was assessed in predicting clinical outcomes.

**Results:**

Out of 182 patients, 11.0% were mechanically ventilated or dead within 28 days. Of patients with MR-proADM >0.87 nmol/L, 21.1% were mechanically ventilated or dead within 28 days, versus 4.5% of those ≤0.87 nmol/L (p< 0.001). The sensitivity, specificity, NPV, and PPV of MR-proADM cut-off of 0.87 nmol/L in predicting mechanical ventilation or death were 75%, 65%, 95%, and 21% respectively, with an AUC of 0.76, outperforming other biomarkers. Specifically, MR-proADM outperformed plasma IL-6 in predicting mechanical ventilation and death.MR-proADM was independently associated with mechanical ventilation or death, ICU admission, prolonged hospitalization beyond day 4, and worsening on the COVID-19 ordinal scale.

**Conclusion:**

MR-proADM functions as a valuable prognostic biomarker in predicting clinical outcomes of patients with COVID-19, outperforming other commonly utilized biomarkers.

**Disclosures:**

**Michael Mansour, MD, PhD**, ThermoFisher Scientific: Grant/Research Support.

